# Fiber Tracts Anomalies in APPxPS1 Transgenic Mice Modeling Alzheimer's Disease

**DOI:** 10.4061/2011/281274

**Published:** 2011-09-06

**Authors:** H. Chen, S. Epelbaum, B. Delatour

**Affiliations:** ^1^CNRS, Laboratoire NAMC, UMR 8620, Université Paris-Sud 11, 91405 Orsay, France; ^2^Department of Neurology, Third Xiangya Hospital, Central South University, Changsha 410013, China; ^3^“Alzheimer's and Prion Diseases” Team, ICM Brain & Spine Institute, UPMC/Inserm UMR-S 975, CNRS UMR 7225, Pitié-Salpêtrière Hospital, 47 Boulevard de l'Hôpital, 75013 Paris, France

## Abstract

Amyloid beta (A*β*) peptides are known to accumulate in the brain of patients with Alzheimer's disease (AD). However, the link between brain amyloidosis and clinical symptoms has not been elucidated and could be mediated by secondary neuropathological alterations such as fiber tracts anomalies. In the present study, we have investigated the impact of A*β* overproduction in APPxPS1 transgenic mice on the integrity of forebrain axonal bundles (corpus callosum and anterior commissure). We found evidence of fiber tract volume reductions in APPxPS1 mice that were associated with an accelerated age-related loss of axonal neurofilaments and a myelin breakdown. The severity of these defects was neither correlated with the density of amyloid plaques nor associated with cell neurodegeneration. Our data suggest that commissural fiber tract alterations are present in A*β*-overproducing transgenic mice and that intracellular A*β* accumulation preceding extracellular deposits may act as a trigger of such morphological anomalies.

## 1. Introduction

Alzheimer's disease (AD) is a highly prevalent neurodegenerative disease accompanied by gradual and irreversible behavioral and cognitive impairments. Brain lesions observed during the course of AD involve two main aspects: extr-acellular amyloid-beta (A*β*) deposition in the senile plaques and intracellular tau accumulation forming neurofibrillary tangles and promoting cytoskeletal disorganization. Current research on AD is largely guided by a dominant pathogenic theory, the so-called amyloid cascade hypothesis [1]. Regularly commented on and amended [2], this model posits accumulation of A*β* in the brain as a key primary event that determines the onset of other brain alterations (e.g., synaptic and neuronal death), finally leading to the dementia. Early onset familial forms of AD are indeed associated with mutations in different genes (amyloid precursor protein (APP) and presenilins 1&2, (PS1&2)) involved in the biosynthesis of A*β*. Dysfunction of these genes is logically thought to alter the rate of APP cleavage, resulting in exaggerated A*β* production. High levels of brain A*β* and associated parenchymal amyloid plaques are key phenotypes described in transgenic mice overexpressing one or more of these mutated genes (see [3] for review). These mice subsequently develop neuropathological alterations and behavioral impairments mimicking AD phenotype [4, 5].

The exact impact of brain A*β* accumulation on clinical symptoms remains to date difficult to decipher, both in AD patients and in animal models of the disease. Clinicopathological correlative analyses have led to mitigated conclusions [6–10], and it is now considered that preplaques A*β* assemblies are the most deleterious species [11] while aggregated insoluble deposits have a reduced pathogenicity [12]. 

In addition white matter anomalies are described in AD patients, presumably in association with cerebrovascular impairments [13], and in transgenic mice modeling brain amyloidosis [14]. These lesions can be detected through conventional postmortem neuropathological examination, but also in vivo by means of dedicated techniques such as diffusion tensor imaging [15, 16], and it has even been proposed that white matter defects are potent diagnostic biomarkers for AD [17, 18]. The integrity of axonal bundles constituting white matter fiber tracts is particularly compromised during the course of AD, even at the very early stages of the disease [19, 20]. Alterations of fiber tracts have obvious functional consequences: disconnection of neural networks occurs following fibers loss, leading to diaschisis and cortical disorganized activity [21, 22]. Also, disruption of myelin in axonal bundles might have deleterious effects on neuronal communication by altering propagation of action potentials and increasing brain energy expenditure (see [23, 24] for reviews). 

White matter alterations in fiber tracts are related to demyelination and also to other axonal morphological defects that can ultimately lead to degeneration and loss of fibers. It has been demonstrated that AD patients show concurrent decreased axonal densities [25] and myelin breakdown [23]. Interestingly, experimental studies in AD animal models have shown that axonal pathology can be driven by A*β* deposition in the brain [26–28]. 

The aim of the present study was to further evaluate fiber tracts changes in a double APPxPS1 mouse transgenic model with aggressive A*β*-related pathology [29–31]. Two commissural fibers tracts (corpus callosum and anterior commissure) that show significant alterations in AD patients [32, 33] were selected and analyzed: axonal integrity was quantified by assessing antineurofilament immunostainings. Myelin density was evaluated by histochemistry using a gold chloride staining that provides high contrast and spatial resolution [34, 35] and that has previously been used to underline myelin alterations in AD transgenics [36]. Age-dependent changes were detected in both fiber tracts of APPxPS1 mice. We tentatively correlated the relationship between these morphological anomalies and A*β* deposition.

## 2. Materials and Methods

### 2.1. Transgenic Mice

Female transgenic APP/PS1 mice (Thy1 APP751 SL (Swedish mutation KM670/671NL, London mutation V717I introduced in human sequence APP751) × HMG PS1 M146L) modeling early onset and progressive cerebral amyloid deposition were used [29, 30, 37–39]. Heterozygous APP/PS1 mice were obtained by crossing APP(+/wt)/PS1(wt/wt) with APP(wt/wt)/PS1(+/+) mice established on a C57bl6/CBA background. Note that, due to the use of homozygous PS1(+/+) mice, no wild-type mice could be generated from the breeding scheme. PS1 littermates were therefore used as control animals. PS1 mice have been described in previous studies to be devoid of neuropathological alterations [30, 37] and hence constitute good controls (age-matched littermates with no overt phenotypes) for analyzing APPxPS1 transgenics. A total of 28 mice were evaluated. Two age groups were analysed: ‘‘young” (2 months: 8 APP/PS1 and 7 PS1 mice) and ‘‘old” animals (24months: 6 APP/PS1 and 7 PS1 mice).

### 2.2. Histology

#### 2.2.1. Brain Processing

Following decapitation, the brains were extracted, fixed in 10% buffered formalin, and stored overnight in a solution of 20% glycerin and 2% dimethylsulfoxide in 0.1 M phosphate buffer for cryoprotection. Brains were subsequently sectioned into 40 ***μ***m thick coronal sections on a freezing microtome. Twelve series of sections ranging from the frontal to the occipital poles were collected and stored at −20°C in cryoprotectant before use.

#### 2.2.2. Myelin Staining

Myelin staining was performed on a batch of serial sections using a protocol derived from [34] with some modifications. Brain sections were rinsed with 0.1 M phosphate buffer (PB) mounted on Superfrost+ slides and dried overnight at 50°C. The slides were then rinsed in 0.02 M phosphate saline buffer (PBS). Two milliliters of freshly prepared 0.2% gold chloride (Carlroth, Karlsruhe, Germany) dissolved in 0.02 M PBS was added on each glass slides to completely cover the section. Incubation was carried out at room temperature (RT) for 2 hours in obscure humid chambers. The tissue was briefly rinsed in water, fixed in 2.5% sodium thiosulfate for 5 minutes, and finally rinsed in running tap water for 30 minutes. Sections were coverslipped with Eukitt after dehydration in graded alcohols and clearing in xylene. 

#### 2.2.3. Axonal Immunostaining

Free-floating sections were rinsed in PB and pretreated with 0.3% H_2_O_2_ in methanol for 10 minutes to block endogenous peroxidase activity. Nonspecific antigenic sites were blocked by 5% goat serum in PBS-Triton X-100 (1 hour at RT). Sections were then incubated overnight at RT with a C-terminal antineurofilament M (145KD) primary antibody (AB1987; Chemicon, Temecula, Calif) diluted 1 : 1000 in serum. The following day sections were incubated in a secondary biotinylated antirabbit antibody solution (Sigma, St. Louis, Mo; 1 : 200, 1.5 hours at RT) and then in an avidin-biotin peroxidase complex (ABC) reagent (Vectastain Elite ABC-Kit, Vector Laboratories, USA, 1 : 800, 1.5 hours at RT). The sections were rinsed 3 × 10 min between all incubation steps. The immunoreaction was visualized with 3,3^'^-diaminobenzidine (DAB) under microscopical control. The reaction was stopped when the signal-to-noise ratio was considered optimal. The sections were finally mounted on superfrost slides, dried, and processed in alcohols/xylene before being coverslipped.

#### 2.2.4. Amyloid Plaques and Intracellular A*β*

One series of section was stained by Congo red according to the standard technique (30 minutes in a 80% ethanol solution saturated with Congo red and sodium chloride) [30, 40]. Another series of sections was immunostained with the primary 4G8 monoclonal anti-A*β* antibody (Signet Laboratories, Dedham, Mass; 1 : 10000, incubation overnight at RT). A high dilution of the biotinylated 4G8 antibody was used to avoid cross-reaction with APP and to solely immunodetect A*β* [41]. Thanks to biotin complexion, no secondary antibodies were used and tissue was directly incubated in the ABC Kit solution before being developed in DAB. 

#### 2.2.5. Evaluation of Neurodegeneration

Staining of degenerating neurons was performed using the Fluoro-Jade B dye [42] with a slightly modified protocol [43]. Glass-mounted sections were passed through absolute ethanol and 75% ethanol followed by a 1-minute rinse in distilled water. Tissue was then incubated in 0.06% potassium permanganate solution for 15 minutes with slight agitation and rinsed before staining in Fluoro-Jade B (Histo-Chem., Jefferson, Ark; 0.001% solution prepared in 0.1% acetic acid; 30 minutes at RT). After extensive rinsing in distilled water, sections were dehydrated, cleared in xylene, and coverslipped.

### 2.3. Quantitative Image Analysis and Statistics

#### 2.3.1. Myelin Densities

All slides were digitized using a Super CoolScan 8000 ED scanner (Nikon, Champigny sur Marne, France) with a 4000 dpi in-plane digitization resolution (pixel size 6.35 *μ*m^2^). Quantification of gold chloride staining was performed, in two regions of interest (corpus callosum and anterior commissure), by relative optical density (ROD) analysis, a method that has already been used to evaluate myelin stainings [44]. In the rostral brain, 3 sections were selected to analyze myelin at the level of the anterior commissure (bilateral countings, i.e., *n* = 6 samples / mouse). For the analysis of myelination of the corpus callosum, 7 sections per animal were selected, spanning the rostrocaudal extent of the region; the ROD analysis was performed on the total corpus callosum volume and also separately in its anterior and posterior parts (see below). 

Measure of mean grey value was semiautomatically performed in the two regions of interest using Photoshop CS2 software (Adobe, Paris, France). ROD was obtained after transformation of mean grey values using the formula ROD = log_10_ (256/(mean grey value)). Values of background staining were taken in adjacent tissues with no myelin staining (upper cortical layers or striatum) and subtracted to get a final densitometric evaluation of myelin stain. 

While 2-month-old APPxPS1 mice were devoid of amyloid plaques, A*β* deposition was very extensive in aged APP/PS1 transgenics. Notably many plaques were observed in the corpus callosum and in the anterior commissure. Obviously the gold chloride negatively stained areas corresponding to plaque occupancy might artificially decrease the local mean grey values. To correct for this bias in old mice, plaques surfaces were manually removed from analyzed regions of interest before processing ROD calculations.

#### 2.3.2. Fiber Tracts Atrophies

**Figure 1:**
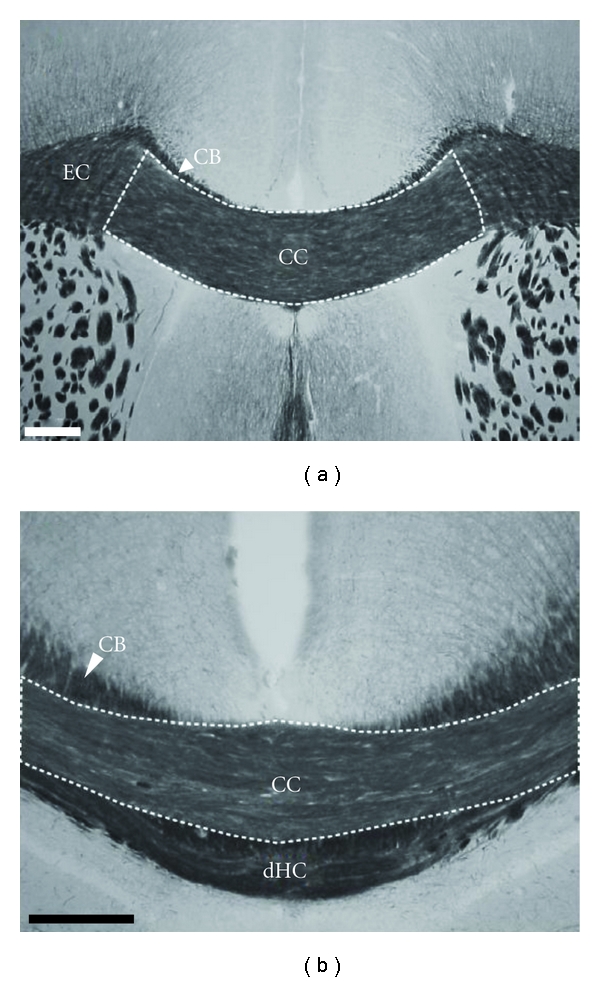
Myelin stain of the corpus callosum. On frontal sections, staining of the myelin using the gold chloride method allows to delineate the perimeter of the corpus callosum (CC) at the rostral (a) and caudal (b) levels. On the basis of axon orientation and staining intensities, adjacent fiber tracts (CB: cingulate bundle; dHC: dorsal hippocampal commissure; EC: external capsule) are clearly outlined allowing accurate visualization of the corpus callosum borders. Scale bars: 200 *μ*m.

The sizes (surface area from coronal sections) of both the corpus callosum and the anterior commissure were evaluated by manually outlining the structures on digitized scans of myelin-stained brain sections (see Figure 1). Three measures of fibers tracts size were taken: (1) in the anterior corpus callosum (sampling between 4.9 and 3.22 mm/interaural reference in the Paxinos and Franklin atlas [45], *n* = 4 sections/mice), (2) in the posterior corpus callosum (3 sections taken between 1.5 and 2.58 mm/interaural reference), and (3) in the anterior commissure (sampling between 4.18 mm and 4.9 mm/interaural reference, *n* = 3 sections analyzed bilaterally/mice). For each region of interest, area size measurement was calculated by summing outlined areas across serial sections.

#### 2.3.3. Axonal Neurofilament Densities

Neurofilament immunostained sections were digitized as described above for myelin-stained sections. Assessment of neurofilament immunoreactivity was performed in the same two regions of interest (corpus callosum, *n* = 7 sections/mouse; anterior commissure, *n* = 3 sections/mouse) by means of ROD analysis.

#### 2.3.4. Extracellular Amyloid Loads in Old Mice

Evaluation of the extracellular amyloid load was only performed in the 24-month-old APP/PS1 mice as almost no Congo red positive deposits were observed in young double transgenic mice. 

Plaques loads were quantified using computer-based thresholding methods [46]. Scans were prepared using Photoshop CS2 to outline selected regions of interest. Images were then processed with ImageJ freeware (Rasband, W.S., ImageJ, US National Institutes of Health, Bethesda, Md, USA, http://rsb.info.nih.gov/ij/, 1997–2006) using a dedicated macrocommand that extracts amyloid deposits from background tissue with no user inputs. Regional amyloid loads were expressed as percent of tissue surface stained by the Congo red dye that corresponds to the proportion of plaques volume. Amyloid loads were evaluated (1) in the whole brain [30], (2) in the rostral isocortex that is richly innervated by axons passing through the anterior corpus callosum, and (3) directly in the two fiber tracts that were investigated in the present study (anterior commissure, corpus callosum).

#### 2.3.5. Intracellular Amyloid Loads in Young Mice

Intr-aneuronal A*β* immunoreactivity was not found in aged mice (as already reported for this mouse line [38]) and was quantified only in the young APPxPS1 mice. Two sections/animals were selected at the level of the frontal cortex, and a semiquantitative analysis, based on a four-point scale, was performed on both hemispheres to evaluate levels of neuronal immunostainings in the cortex: 0: no obvious positive staining; (1): weak intracellular staining; (2): moderate staining; (3): strong staining. For each animal semi-quantitative analyses were hence performed in four fields (2 slices; bilateral countings).

To evaluate the connection properties of A*β*-containing neurons and their possible participation to commissural tracts we performed an axonal tracing study in two wild-type mice (3 month-old): biotinylated dextran amine (BDA), an anterograde tracer, was stereotactically injected in the cortical regions where A*β*-positive neurons were concurrently observed in APPxPS1 transgenics, allowing fine visualization of cell bodies and of neurites. Surgery and immunolabeling of BDA stained axons were performed using standard protocols as previously described [47, 48].

#### 2.3.6. Statistics

Student's *t*-tests were performed using Statistica 7 (StatSoft, Inc., Tulsa, USA). Results were considered statistically significant at *P* < 0.05.

## 3. Results

### 3.1. Altered Volumes of Fiber Tracts in APPxPS1 Mice

Gold chloride myelin staining, as compared to standard stains (e.g., HE or Nissl stains), allowed to precisely outline the area of the corpus callosum. In particular, delineating the corpus callosum from adjacent white matter tracts (e.g., cingulate bundle, dorsal fornix, and dorsal hippocampal commissure) was greatly facilitated by the myelin staining (Figure 1). Lateral limits of the corpus callosum and borders of the external capsule were identified by a horizontal-to-vertical shift in fiber orientation. Also, the anterior commissure was easily identified and outlined in the gold chloride-stained sections (see Figure 5(b)).

#### 3.1.1. Young Mice

**Figure 2:**
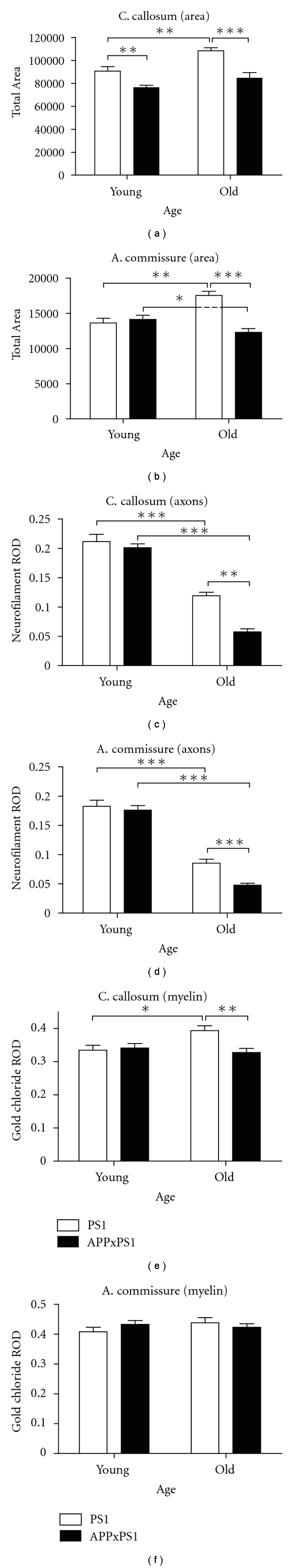
Summary of quantitative morphological analysis. The analysis was focused on the corpus callosum (left column) and on the anterior commissure (right column). Morphological data were collected in old and in young mice from PS1 and APPxPS1 genotypes. (a)-(b). Size of fiber tracts (pixels). (c)-(d). Relative optical densities (ROD) of neurofilament M145Kd (axons) immunostainings. (e)-(f). Relative optical densities of gold chloride (myelin) stainings. See text for details. All measures are illustrated by means + SEM in each experimental groups.  *P < .05,   **P < .01,  ***P < .001.

The size of the anterior commissure was similar in 2-month-old PS1 mice and in age-matched APPxPS1 transgenics (*t*(13) = −0.58; Figures 2(b) and 5(b)). On the contrary, the corpus callosum size was significantly decreased in young APPxPS1 as compared to controls (*t*(13) = 3.501, *P* < .005; Figures 2(a) and 5(a)). Subregional analysis indicated a significant reduction in the size of the rostral corpus callosum of young APPxPS1 mice (*t*(13) = 3.743, *P* < .005) while there was no difference between genotypes in the size of the posterior corpus callosum (*t*(13) = 0.136, ns).

#### 3.1.2. Old Mice

With aging, a significant increase in the size of axonal bundles was observed in PS1 control mice (corpus callosum: *t*(12) = 3.858, *P* < .005; anterior commissure: *t*(12) = 4.275, *P* < .005). From pilot studies, we have observed the same developmental traits in wild-type C57bl6 mice (increased volumes of the corpus callosum and of the anterior commissure with aging; data not shown) precluding the possibility that the PS1 transgene by itself triggered abnormal (increased) fiber tract growth in PS1 transgenic mice. 

On the other side, age-dependent enlargement of commissural bundles was clearly not evidenced in the double APPxPS1 transgenics: in this genotype, the size of the corpus callosum remained constant between 2 and 24 months (*t*(12) = 1.850, ns) and the surface area of the anterior commissure even undergoes slight atrophy with aging (*t*(12) = 2.284, *P* < .05). Hence, strong differences between genotypes were observed in 24-month-old mice with APPxPS1 transgenics showing, in comparison to PS1 controls, decreased white matter surface areas. This was observed at the level of the anterior commissure (*t*(11) = 6.388, *P* < .0001) and of the corpus callosum (total corpus callosum: *t*(11) = 4.653, *P* < .001; anterior part: *t*(11) = 5.404, *P* < .0005). The only posterior region of the corpus callosum did not show significant atrophy in old APPxPS1 mice (*t*(11) =1.492, ns).

### 3.2. Potentiation of Axonal Neurofilament Loss in Old APPxPS1 Mice

**Figure 3:**
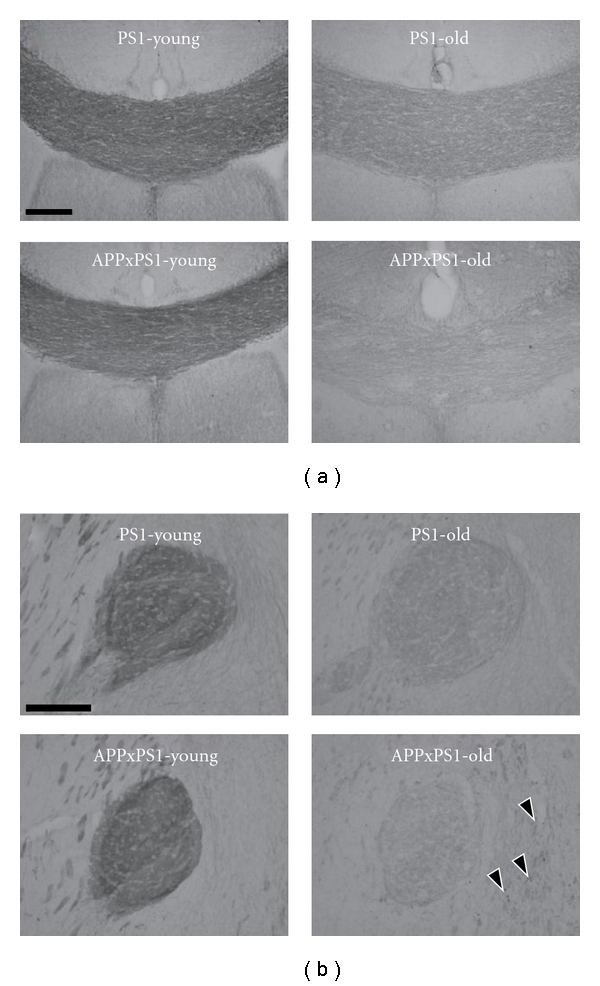
Representative illustration of neurofilament immunostainings. Immunodetection of axonal neurofilaments is illustrated at the level of the corpus callosum (a) and anterior commissure (b) in both young and old PS1 and APPxPS1 transgenics. See text for details concerning age and genotype effects. Black arrow heads point to positive axonal enlarged varicosities in old APPxPS1 mice. Scale bars: 100 *μ*m.

Densities of axonal neurofilaments in the corpus callosum and in the anterior commissure were quantified using ROD analysis of immunostainings. In 2-month-old mice axonal densities were similar in both genotypes whatever the fiber tract considered (all *P*'s >.35). With aging, a severe decrease in neurofilament density was observed, both in APPxPS1 and PS1 mice, in the corpus callosum (Figures 2(c) and 3(a)) and in the anterior commissure (Figures 2(d) and 3(b)) (all *P*'s <.0001). Age-related reduction of neurofilament density was, however, largely more prominent in old APPxPS1 mice than in age-matched PS1 controls. This accelerated loss of axonal neurofilaments in old APPxPS1 mice was further confirmed by statistical analysis in the different subregions of the corpus callosum (APPxPS1 < PS1: all *P*'s <.0001) and 2) and also at the level of the anterior commissure (*t*(10) = 4.16; *P* < .005).

### 3.3. Abnormal Myelination in Old APPxPS1 Mice

ROD analysis of gold chloride stainings in 2-month-old mice indicated comparable myelin densities in PS1 and APPxPS1 transgenics (corpus callosum: *t*(13) = 0.318, ns, Figures 2(e) and 5(a); anterior commissure: *t*(13) = 1.277, ns, Figures 2(f) and 5(b)). 

The myelin density of the anterior commissure was not affected by aging (PS1: *t*(12) = 1.292, ns; APPxPS1: *t*(12) = 0.556, ns), and myelin densities in this fiber tract were similar in 24-month-old PS1 and in age-matched APPxPS1 mice (*t*(11) = 0.679, ns). 

On the other hand, an increase of the myelination of the corpus callosum was observed when comparing 2-month- and 24-month-old PS1 mice (*t*(12) = 2.823, *P* < .05). Noticeably, this increase in myelin density was significant in the rostral corpus callosum (*t*(12) = 4.171, *P* < .005) but not in its posterior part (*t*(12) = 0.461, ns). Contrarily to PS1 mice, such an age-related increase of callosal myelination was not observed in APPxPS1 mice (*t*(12) = 0.7, ns). Consequently decreased myelin staining was evidenced in 24-month-old APPxPS1 mice when compared to old PS1 controls (total corpus callosum: *t*(11) =3.332, *P* < .01). Differences between genotypes were further confirmed at the level of the anterior corpus callosum (*t*(11) = 3.512, *P* < .005), while no difference between PS1 and APPxPS1 mice were evidenced in more caudal regions (*t*(11) = 1.9; ns).

**Figure 4:**
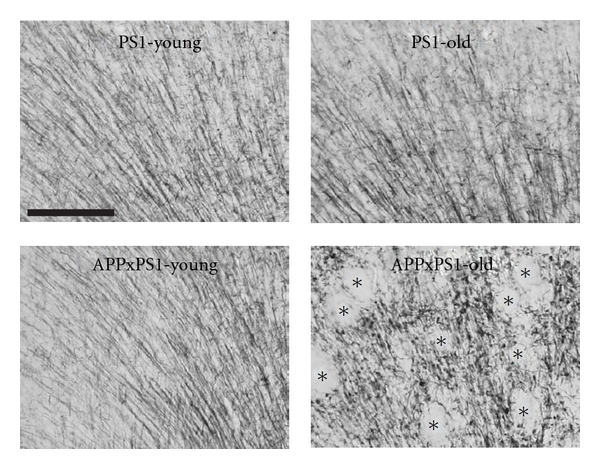
Myelin stains in the cingulate cortex. In young PS1 and APPxPS1 mice myelinated axons in the cingulate cortex have a radial organization that is preserved in old PS1 mice but is severely disorganized in old APPxPS1 transgenics (lower right photo: optically empty areas correspond to amyloid plaques and are identified by black asterisks). Scale bar: 100 *μ*m.

**Figure 5:**
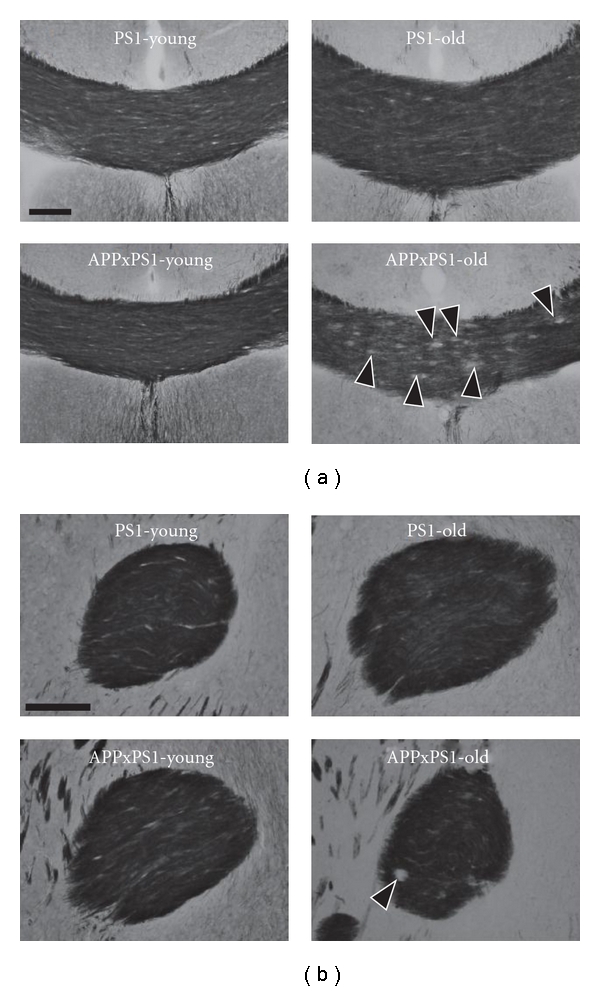
Representative illustration of myelin stains of fiber tracts. Myelin (gold chloride) stains are illustrated at the level of the corpus callosum (a) and anterior commissure (b) in both young and old PS1 and APPxPS1 transgenics. See text for details concerning age and genotype effects. Black arrow heads ((a) lower right photo) point to circular unstained areas corresponding to amyloid plaques in the corpus callosum of old APPxPS1 mice. Scale bars: 100 *μ*m.

Qualitative examination of myelin-stained sections was then performed in old APPxPS1 mice. No evidence of myelin breakdown (debris) was found in the large myelinated bundles of the corpus callosum. However, in comparison to control animals, myelin appeared to be fragmented in the isocortex and hippocampus of APPxPS1 mice (Figure 4). Myelin material was often detected under the form of small tortuous segments with bead-like varicosities. These morphological anomalies, absent in young APPxPS1 mice, were found at the vicinity of A*β* deposits but also in the parenchyma in areas distant from plaques. 

### 3.4. Relationship with A*β* Pathology and with Neurodegeneration

**Figure 6:**
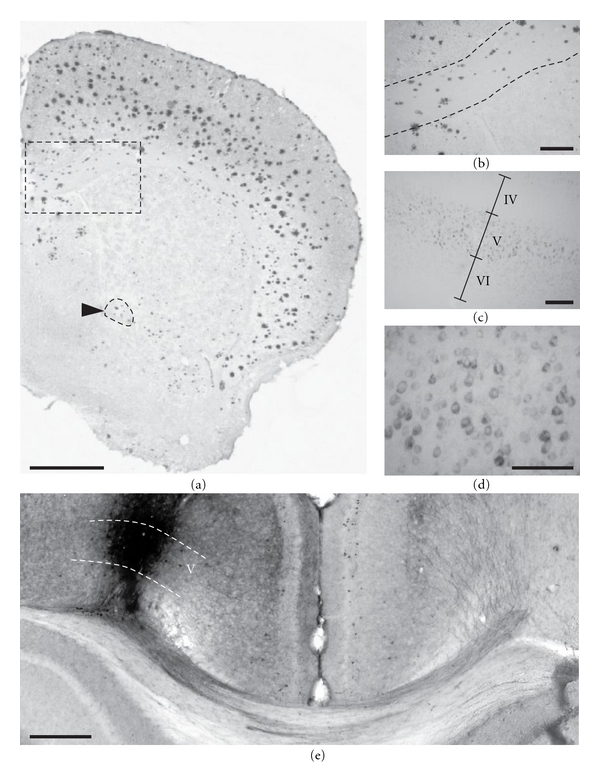
A*β* pathology in APPxPS1 mice. 24-month-old APPxPS1 mice display heavy cortical amyloid burden as evidenced by Congo red staining (a). Congophilic plaques were also observed in white matter tracts such as the corpus callosum (outlined rectangular area in magnification in (b)) and the anterior commissure (outlined in a and pointed by a black arrow head). In young APPxPS1 mice dense core amyloid plaques were virtually absent but strong A*β* intraneuronal accumulation, as revealed by 4G8 immunostainings, was detected in infragranular cell layers ((c), magnified view in (d)). (e) Biotinylated dextran amine, an anterograde tracer, was injected in the deep layers of the isocortex (left hemisphere) at the locus where A*β*-containing neurons were found in APPxPS1 transgenics. These neurons send their axons through the corpus callosum and cingulum bundle, cross the midline, and innervate the opposite (right) hemisphere. Scale bars: 1000 *μ*m (a), 100 *μ*m ((b), (c), and (d)), and 500 *μ*m (e).

Congo red positive aggregates were detected and quantified in the anterior commissure (mean load= 2.8%; min = 1.6%; max = 6%) and in the corpus callosum (mean load = 2.2%; min = 1.6%; max = 2.7%) of old APPxPS1 mice (Figures 6(a) and 6(b)). Correlative analysis did not reveal significant associations between local amyloid loads in fiber tracts and decreased neurofilament immunoreactivity/myelin densities (all *P*'s >.111). Also there were no correlations between the sizes of the corpus callosum/anterior commissure and total or cortical amyloid loads (all *P*'s >.196).

In addition to amyloid plaques loads, intraneuronal A*β* was semiquantitatively assessed in the isocortex of young APPxPS1 mice. As expected from previous observations, positive labeling was detected using the 4G8 antibody in a subset of cells. Staining was mainly observed in deep cortical layers (V) involving a distinctive band of large pyramidal cells (Figures 6(c) and 6(d)). There were no direct correlations between levels of intracellular A*β* that may significantly vary from one animal to the other (mean = 7.8; min = 4.5; max = 11.5) and neurofilament and myelin densities (all *P*'s >.119). Interestingly, tracing experiments underlined that the deep layers of the cortex, where specifically A*β*-positive neurons were observed in the double transgenics, are the source of dense callosal projections (Figure 6(e)). Therefore, although no tight correlations were stressed between the density of intracellular A*β* and the magnitude of axonal anomalies, we evidenced that callosal fibers still originate from a subpopulation of neurons overproducing toxic A*β* species.

The Fluoro-Jade B dye was used to assess neurodegeneration in APPxPS1 mice. No positive neurons were detected in the studied animals (data not showed). In particular no degenerating neurons were observed in the cortical layers with high densities of A*β*-positive neurons (see above). Only the core of amyloid deposits and the surrounding degenerating dystrophic neurites as well as reactive astrocytes were detected with Fluoro-Jade B in old APPxPS1 mice (see [49, 50] for similar observations).

## 4. Discussion

Neuroimaging assessment repeatedly underlined white matter changes in AD patients. Evidence from diffusion tensor imaging of fiber tracts suggests for instance that during the course of the disease there is a loss of barriers that restrict water motion and tissue anisotropy of white matter. These observations have obviously a neuropathological counterpart: anomalies of diffusion could reflect either loss of axons, demyelination, and/or oligodendroglial pathology. To date only a few studies have investigated the source of white matter/fiber tracts impairments in AD patients and in aged nondemented subjects [51, 52]. Recent attempts to decipher the origin and mechanisms of such morphological alterations have been performed in animal models of the disease [36, 53, 54] but a systematic screening of fiber tracts anomalies related to AD pathology is still lacking in these models.

The goal of our work was therefore twofold: (1) to assess in an AD mouse model the severity of fiber tracts alteration across aging and (2) to refine the understanding of their histological substratum and in particular to evaluate the relation between A*β* peptide deposition and the occurrence of the white matter anomalies.

### 4.1. Fiber Tract Atrophies in APPxPs1 Mice

Our quantitative analysis underlines the decreased size of forebrain fiber bundles in old APPxPS1 mice displaying concurrent severe brain amyloidosis. Volume reductions were observed in the corpus callosum, as already reported in AD transgenics [14, 30, 55] but also, as a novel finding, at the level of the anterior commissure. These anomalies do not purely mimic an age-related atrophy and might also reflect a lack of normal development of fiber tracts in APPxPS1 mice (see also [30] for similar conclusions). 

As mentioned above, atrophy of the corpus callosum has previously been reported in AD transgenic mouse models but more interestingly is also classically depicted in AD patients (e.g., [33]). We found that atrophy of the corpus callosum, in our APPxPS1 transgenics, largely predominates in its anterior part. Although not constantly reported, similar findings concerning regional atrophies of the corpus callosum have been described in human patients [21, 56]. In addition to callosal atrophy we also evidenced, in old APPxPS1 mice, a reduction in size of the anterior commissure that might find a parallel with recent observations in AD patients [32]. 

Supporting size reduction of fiber tracts we evidenced a very strong decrease of axonal neurofilament densities in the double transgenics, outclassing the “normal” loss of neurofilament we observed in aged control PS1 mice (and in wild-type mice; data not shown). We also substantiated a significant myelin breakdown in APPxPS1 mice. The loss of myelin in aged APPxPS1 mice appears however to be limited in comparison to the high and accelerated decrease of neurofilament densities these mice undergo. As evidenced by correlative analysis, there were no strict linear relationships between fiber tracts atrophies and neurofilament or myelin markers. For instance, we found a reduced size of the corpus callosum in young APPxPS1 mice while these animals showed the same neurofilament and myelin densities as control PS1 mice—such observations emphasizing similar densities of axonal markers in an overall reduced volume of tissue might indeed testify for an early net loss of callosal fibers in young APPxPS1 mice. Also we found that fiber tracts continue to grow and pursue their myelinisation in normal mice between 2 and 24 months of age while neurofilament densities in the same mice concurrently display a 50% drop—this observation can be explained (1) by a progressive enlargement of myelin sheets with aging that will maximize space around individual axons and consequently decrease their density and (2) by a concurrent diminution of the expression of axonal neurofilament proteins that has previously been described in aged animals [57]. 

### 4.2. Substratum of Fiber Tract Anomalies: Altered Myelinisation

We have investigated various sources of neuropathological alterations that could explain fiber tracts atrophies in our APPxPS1 model. It has been claimed that a demyelination process is observed during the course of AD and is a trigger in the physiopathogeny of this disease [23, 24]. It has even been proposed that myelin breakdown in AD initiates a vicious circle as release of iron after demyelination might promote A*β* production and toxicity which in turn affects myelin integrity [58]. In the present study we indeed observed qualitative anomalies of axonal myelination in old APPxPS1 mice accumulating A*β*. Similar observations, that might deserve confirmation and further characterization at the ultrastructural level using electron microscopy, have been recently reported in AD Tg2576 and APPxPS1 mice but only in the neuropil in close association with amyloid plaques [54]. In our study myelin alterations, including fragmentation and spheroid-like pathological enlargements, were observed in axons passing through the gray matter, even at distance of amyloid deposits, but not easily in dense fiber tracts under light microscopy. At the quantitative level, the overall myelination of the corpus callosum was found to be significantly decreased in old APPxPS1 mice. However no differences were observed between genotypes at the level of the anterior commissure, suggesting that loss of myelin is heterogeneous and does not affect similarly all axonal bundles. Actually, myelination kinetics during ontogenesis might considerably vary from one brain region to the other [44, 59]. It is hence possible that brain areas and fiber tracts with late or early myelination are differentially impeded during (pathological) aging [60, 61]. In addition, it is known that the corpus callosum, in terms of phylogeny, is a neural pathway that is only found in placental mammals while the anterior commissure is a more ancestral axonal tract [62], adding supplementary differences between the two fiber bundles that could explain regional discrepancies in age-dependent (de)myelination processes. Interestingly, it has been shown that oligodendrocytes have a reduced myelin turnover in regions lately myelinated during ontogenesis [63]. This would imply a diminished capacity for myelin repair in these regions, especially under challenging conditions (oxidative stress, excitotoxicity, inflammation, etc.) because oligodendrocytes are a strikingly vulnerable population of cells [23]. Toxicity of A*β* on oligodendrocytes has been evidenced in several reports [36, 64] with effects ranging from mitochondrial dysfunction to apoptosis and cell death. The involvement of oligodendrocytes in the myelin anomalies depicted in our APPxPS1 transgenics would deserve further analysis. In particular, it would be interesting to document possible anomalies at the level of myelin basic proteins. 

### 4.3. Substratum of Fiber Tract Anomalies: Axonal Pathology

Decreased sizes of the corpus callosum and of the anterior commissure in APPxPS1 mice might be related to defects in fiber myelination but, according to our observations, can also be linked to an accelerated loss of axonal neurofilaments, possibly reflecting loss of fibers. 

We have previously reported vascular alterations (vessel voids and reduction of vessel lengths) by means of in vivo magnetic resonance angiography in the APPxPS1 transgenic mouse model used in the present study [65]. These lesions, closely associated with amyloid angiopathy, could contribute to the described axonopathies [66].

Anomalies in regulatory proteins involved in axonal structural plasticity during AD could also be causative [67] but remain to be firmly established in our transgenic mouse model.

Axonopathy has previously been reported in the APPxPS1 mouse line but was originally evidenced at the spinal level [31]. Our results highlight that axonal pathology in APPxPS1 mice also concerns forebrain fiber tracts. Interestingly, it has been demonstrated that AD patients sustain axon loss [25, 68] but the magnitude of the effects we reported (large drop in neurofilament densities, first in aged PS1 control mice and second, greatly accentuated, in aged double APPxPS1 transgenics) has not been reported up to now to the best of our knowledge. Bussières and collaborators [69] have demonstrated that neurons from AD brains expressing neurofilament proteins with medium- and heavy- molecular-weight subunits (SMI-32 immunoreactive neurons) constitute an exquisite vulnerable subpopulation of cells during disease process. These neurons have a specific laminar distribution in the cortex compatible with their participation to corticocortical connections relying on fiber tracts and axonal fasciculi. Back to our experimental data, it would be appealing to speculate that loss of axonal neurofilaments in the anterior commissure and corpus callosum indeed implies pathological events at the level of efferent neurons in specific brain regions (e.g., isocortex area for callosal neurons). We did not find however any evidence of cell degeneration in old APPxPS1 mice (see also [50] for similar report). On the other side we cannot totally preclude that APPxPS1 mice have decreased neuronal densities in projections regions resulting from earlier insults, with no overt signs of active degeneration in the oldest animals. A substantial (30%) loss of neurons has been described in the hippocampus of old APPxPS1 mice [37, 70]. It is not yet known if a similar loss occurs in isocortical areas and further analysis will be required to ascertain this point. 

### 4.4. Substratum of Fiber Tract Anomalies: Role of A*β*-Positive Lesions

In accordance with previous studies (e.g., [37, 71]), we did not find evidence of a clear pathogenicity of aggregated (Congo red positive) insoluble A*β* deposits. The density of amyloid plaques did not predict, in old APPxPS1 mice, the magnitude of fiber tracts anomalies these mice sustained. In a previous study we evidenced that axon terminals of cortico-cortical fibers endorse degeneration when contacting amyloid plaques [26] but this local synaptotoxicity was not associated with clear retrograde degeneration. In another study of Jantaratnotai [27] axonal damage and demyelination were reported following in vivo A*β* injections in the corpus callosum. However these effects, obtained in a context of sudden and potentiated toxicity, cannot be considered as conclusive. Furthermore, Stokin and collaborators [72] in a recent study have confirmed that axonal defects induced by APP/PS1 mutations are not directly caused by A*β* overproduction and amyloid accumulation. These late results strengthen our observations. 

While amorphous insoluble amyloid deposits do not appear to be deleterious per se, our results support the hypothesis of a pathogenicity of A*β* when it accumulates intraneuronally [73, 74]. As described by others [75], young APPxPS1 mice display intracellular A*β* accumulation, several weeks before the occurrence of amyloid plaques. In the present study, A*β*-positive neurons were observed, in 2-month-old APPxPS1 mice, in specific layers of the cortex (mostly layer V). In primates, including humans, it is known that pyramidal neurons projecting through the corpus callosum belong to lamina III and V of the association cortex [76–78]. In mice also the origins of callosal afferents have been traced back to cell bodies in layer V [79]. By means of anterograde tracings we confirmed that the A*β*-containing neurons in the deep layers of the cortex participate to commissural fibers passing the midline through the corpus callosum. One may infer from these data that, early during aging, APPxPS1 mice accumulate A*β* in a subset of cortical neurons that later become dysfunctional, develop axonal pathology (loss of neurofilament immunoreactivity and of myelin), possibly through a dying-back mechanism [80], and eventually die [41], leading to a definite loss of axons in brain commissures. 

To summarize, we found, in APPxPS1 mutant mice modeling AD brain amyloidosis, evidence of fiber tracts atrophies. These anomalies are potentiated in aged mice but might partly be explained by some neurodevelopmental defect. Axonal pathology developed by APPxPS1 mice does not appear to be caused by amorphous insoluble amyloid deposits but intracellular A*β* that accumulates in projecting neurons could be a causative factor. The atrophies in fiber bundles we depicted in APPxPS1 mice resemble those described in AD patients and are concomitant with a loss of axonal neurofilaments and a myelin breakdown. These results renew the importance of white matter impairments in Alzheimer's disease as these anomalies, with clear functional impact, can be reproduced in mice overexpressing A*β*, a molecular actor that is the principal target for current therapeutical assays in this devastating disease.
